# Feasibility of shear wave elastography for assessing steatosis in early-stage non-alcoholic fatty liver disease

**DOI:** 10.1371/journal.pone.0324637

**Published:** 2025-05-29

**Authors:** Hui Jiang, Chuan Qin, Yue-Mei Xu

**Affiliations:** 1 Department of Medical Ultrasound, Jinshan Hospital of Fudan University, Shanghai, China; 2 Department of Medical Ultrasound, The Central Hospital of Karamay, Karamay, China; 3 Department of Traditional Chinese Medicine, Jinshan Hospital of Fudan University, Shanghai, China; King Abdulaziz University, SAUDI ARABIA

## Abstract

Non-alcoholic fatty liver disease (NAFLD) is characterized by hepatic fat accumulation with varying degrees of severity. This study aimed to evaluate the feasibility of shear wave elastography (SWE) for assessing the grade of liver steatosis in early-stage NAFLD without fibrosis. A total of 260 subjects were categorized into four groups of G0 (n = 81), G1 (n = 63), G2 (n = 54), and G3 (n = 62). Conventional ultrasound and point SWE examinations were used to assess the grade of liver steatosis in varies degrees and compared with MRI-proton density fat fraction (MRI-PDFF), which was used to quantify hepatic fat content. SWE demonstrated high reproducibility across all groups with interclass correlation coefficients ranging from 0.80 to 0.94. The correlation between SWE and MRI-PDFF were 0.68, 0.71, 0.68 and 0.53 for G0-G3 NAFLD. For conventional ultrasound, the diagnostic performance were 0.77 (95% CI: 0.71–0.83), 0.76 (95% CI: 0.71–0.82), 0.76 (95% CI: 0.70–0.83), for G0 versus G1-3, G0-1 versus G2-3, and G0-2 versus G3. For SWE, the diagnostic performance were 0.88 (95% CI: 0.84–0.92), 0.86 (95% CI: 0.81–0.90), and 0.81 (95% CI: 0.75–0.87), for G0 versus G1-3, G0-1 versus G2-3, and G0-2 versus G3. The SWE showed better diagnostic performance than conventional ultrasound in G0 versus G1-3 (p = 0.003), G0-1 versus G2-3 (p = 0.002), but not in G0-2 versus G3 (p = 0.262). SWE is a reliable tool for assessing the grade of liver steatosis, which could be a valuable tool for monitoring and grading NAFLD in early-stage.

## Introduction

Non-alcoholic fatty liver disease (NAFLD) represents a spectrum of liver conditions characterized by the accumulation of fat in the liver in individuals who do not engage in significant alcohol consumption [1]. NAFLD is increasingly recognized as a major public health issue worldwide particularly in regions with high rates of obesity and metabolic disorders [2]. NAFLD is associated with type 2 diabetes, hypertension, and cardiovascular disease, which is a leading cause of liver-related morbidity and mortality [3].

The reference standard for diagnosing and staging NAFLD has traditionally been histological biopsy through liver [4]. However, liver biopsy is an invasive procedure with high costs and inconsistent results due to sampling issues. Furthermore, patients are difficult to undergo repeated biopsies for monitoring liver steatosis progression over time. Thus, there is growing interest in non-invasive methods for evaluating NAFLD.

Traditional imaging techniques such as ultrasound or computed tomography (CT) can detect fat in the liver. However, they often lack the sensitivity and specificity to assess the severity of liver fat accumulation. MRI-proton density fat fraction (MRI-PDFF) can be used as another non-invasive imaging techniques for quantifying hepatic fat content [5]. MRI-PDFF measures the proportion of fat in the liver by assessing the signal intensity difference between fat and water [6]. Previous studies showed that MRI-PDFF provides a direct and quantitative assessment of liver fat content, which has been validated against histological liver biopsy [7,8].

However, MRI-PDFF cannot provide real-time measurements and arising high cost problem during continuous monitoring, particularly when monitoring the fluctuating progression of NAFLD and tailoring individualized therapeutic interventions. Shear wave elastography (SWE) is a relatively new and non-invasive ultrasound-based imaging technique, which utilizes the propagation speed of shear waves within liver tissue to estimate tissue stiffness [9,10]. Previous studies suggests that SWE could serve as an adjunct diagnostic methods in evaluation of steatosis [11]. Interestingly, studies reported that the accuracy of SWE measurements of liver fat may be interfered in the presence of fibrosis [12]. Wu et al. reported that SWE measurements correlate with liver steatosis in rat NAFLD models in absence of fibrosis [13]. These studies suggest that SWE could be a useful tool for assessing NAFLD in early-stage. However, to the best of our knowledge, no SWE studies in evaluation varies grade of steatosis in early-stage NAFLD has been carried out.

We hypothesize that SWE could be used for assessing liver steatosis in early-stage NAFLD. This study aims to extend these findings by providing a feasibility and reliably analysis of SWE to assess the severity of liver fat accumulation in NAFLD, particularly when fibrosis is not yet present.

## Materials and methods

### Ethical considerations

This study adhered to the ethical guidelines established by the Declaration of Helsinki and received approval from the Institutional Review Board of Jinshan Hospital (JIEC 2022-S41). All participants provided written informed consent before their inclusion in the study. Patients or the public were not involved in the design, or conduct, or reporting, or dissemination plans of our research.

### Data collection

This prospective study included suspect NAFLD patients between 1^st^, September 2022 and 1^st^ August 2023. The inclusion criteria were as follows: individuals aged between 18 and 75 years, no significant alcohol consumption (less than 20 grams per day for women and 30 grams per day for men). The exclusion criteria were as follows: patients with any other liver diseases such as viral hepatitis, autoimmune disorders, or cirrhosis, pregnancy, or significant liver fibrosis [aspartate aminotransferase-to-platelet index ratio (APRI index) beyond 2, fibrosis score based on four factors index (Fib-4 index) below 1.45 or NAFLD fibrosis score (NFS) beyond 0.676]. Each participant underwent imaging with conventional ultrasound, SWE and MRI-PDFF to measure liver fat content on the same day, respectively.

### Clinical laboratory tests

Clinical laboratory tests were performed after a minimum 8-hour fasting period on the same day before imaging examination. Participants underwent a comprehensive clinical assessment, which included a thorough medical history and physical examination. Blood samples were collected to evaluate liver function and lipid profile including alanine aminotransferase (ALT), aspartate aminotransferase (AST), and gamma-glutamyl transferase (GGT), alkaline phosphatase (ALP), and total bilirubin (TB), total cholesterol (CHOL), triglycerides (TG), low-density lipoprotein (LDL), high-density lipoprotein (HDL), Platelet (PLT), and albumin (ALB).

The serological diagnostic models of APRI index, Fib-4 index and NFS was applied with the calculation formulas as follows: APRI = AST (U/L)/PLT (10^9^/L); Fib-4 = [age (y) × AST (U/L)]/[PLT (10^9^/L) × ALT (U/L)¹/²]; NFS = −1.675 + 0.037 × age (y) + 0.094 × body mass index (BMI, kg/m²) + 1.13 × whether there is impaired glucose tolerance or diabetes (yes is 1, no is 0) + 0.99 × AST/ALT ratio – 0.013 × platelets (10⁹/L) – 0.66 × albumin (g/dL).

### Ultrasound and shear wave elastography examination

Ultrasound examination was carried out using ultrasound systems (ACUSON Sequoia, Siemens, German) with a C5-1 detector with frequency range 3.55–5.55 MHz. The patients were in a supine position with the right arm elevated above the head to facilitate optimal imaging access to the liver. The probe was placed perpendicular to the 7th, 8th, and 9th intercostal spaces on the right anterior axillary line to avoid interference from rib acoustic shadows. The right liver lobe S5 and S8 and the left liver lobe S3 and S4 were displayed. The patients were asked to hold the breath, wait for the image to stabilize, when collecting the data. During the detection process, large blood vessels and gallbladder were avoided. The influence of cardiac and respiratory movements on the examination were also avoided.

First the severity of the NAFLD was evaluated under conventional ultrasound [14]. Four degrees was recorded: normal, intermediate (minimal increase in liver echogenicity, normal visualization of intrahepatic vessels), moderate (moderate increase in liver echogenicity, slightly impaired visualization of intrahepatic vessels), severe (marked increase in liver echogenicity, poor or no visualization of intrahepatic vessels).

Then the B-mode image was optimized, and the point SWE mode was switched. The sampling frame depth was 3–6 cm, and at least 2 cm below the liver capsule. The sampling frame was kept in the center of the image. The interquartile range/median ratio of the obtained SWE velocity value was controlled lower than 30%. The region of interesting (ROI) used the default values built into the instrument. The system automatically processed and recorded the SWE velocity value. Each patient was required to repeat the measurement 10 times (5 from the right/left lobe, respectively), and the mean value was calculated. When focal fatty infiltration of liver was identified on the image, only the value of the fatty infiltration lobe was recorded.

The procedure was first performed by a radiologist (with 5 years experience in abdominal ultrasound). Then the SWE were repeat recorded by a second radiologist (with 15 years experience in abdominal ultrasound). Interclass correlation coefficient (ICC) was used for stability analysis of SWE in different hepatic steatosis groups.

### MRI-proton density fat fraction examination

MRI-PDFF was performed using 3T MRI scanners (UHR780, United Imaging, China). The built-in liver fat quantitative scanning sequence was used. The patient was in the supine position. The upper abdomen of the subject was scanned. The imaging technique employed a multi-echo Dixon method to quantify liver fat content by separating water and fat signals. This approach provides a precise, quantitative assessment of liver fat, expressed as a percentage. The scanning parameters were as follows: TR = 10.8 ms, TE1 = 1.72 ms, TE2 = 3.25 ms, TE3 = 4.78 ms, TE4 = 6.31 ms, TE5 = 7.84 ms, TE6 = 9.34 ms, slice thickness = 6.0 mm, number of slices = 24. MRI-PDFF was measured on the automatically generated liver fat fraction map. Six ROIs of 10 cm^2^ was drew on the right and left lobe of the liver were measured, respectively. The average value of the six ROIs was taken. The large blood vessels and gallbladder of the liver were avoided as much as possible during the measurement. When focal fatty infiltration of liver was identified on the image, only the value of the fatty infiltration lobe was recorded. The measured MRI-PDFF is divided into four levels [15]: G0 level PDFF < 5.2%; G1 level 5.2% ≤ PDFF < 11.3%; G2 level 11.3% ≤ PDFF < 17.1%; G3 level PDFF ≥ 17.1%.

### Statistical analysis

The statistical analysis was performed using R (Version 4.4.0). Continuous variables were presented as means ± standard deviation (SD). Categorical variables were expressed as counts and percentages. To compare differences between groups, one-way analysis of variance (ANOVA) or the Kruskal-Wallis test was used for continuous variables based on whether normally distributed of the data. While the Chi-square test was applied for categorical variables. To assess the correlation between SWE and MRI-PDFF, Pearson’s correlation or Spearman’s rank correlation were used. Receiver operating characteristic (ROC) curves were generated to evaluate the diagnostic performance of SWE for detecting varying degrees of liver fat contents. The optimal cutoff values for SWE were determined by analyzing sensitivity, specificity, positive predictive value (PPV), and negative predictive value (NPV) at different thresholds. A p-value of <0.05 was considered statistically significant for all tests.

## Results

### Demographic and clinical parameters

**Fig 1:**
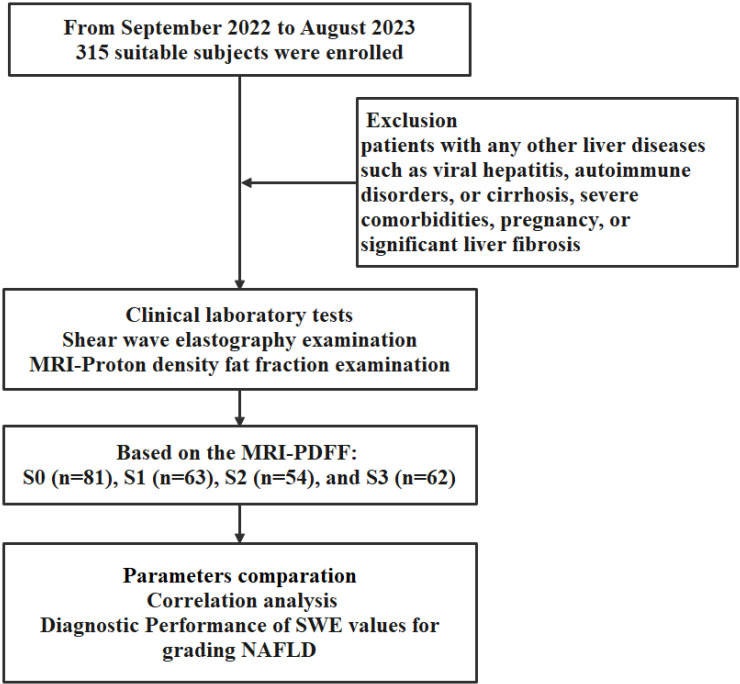
The work flow of this study for non-alcoholic fatty liver disease (NAFLD) assessment using point shear wave elastography (SWE) and MRI-proton density fat fraction (MRI-PDFF).

The work flow of this study is shown in Fig 1. The study included 315 patients. After excluded 55 patients exhibited liver fibrosis, 260 patients were classified into four groups based on the MRI-PDFF: G0 (n = 81), G1 (n = 63), G2 (n = 54), and G3 (n = 62). The gender distribution varied by severity, with 50.6% females in G0, 34.9% in G1, 31.5% in G2, and 27.4% in G3. The proportion of females significantly decreased as liver steatosis worsened. No significant age differences were observed between groups. The baseline characteristics and laboratory values of participants in each group are summarized in Table 1.

**Table 1:** The Baseline Characteristics and Laboratory Values of Participants in Each Group.

	G0	G1	G2	G3	p^#^	p^$^	p^*^
(N = 81)	(N = 63)	(N = 54)	(N = 62)
Gender							
Female	41 (50.6%)	22 (34.9%)	17 (31.5%)	17 (27.4%)	0.004	0.024	0.09
Male	40 (49.4%)	41 (65.1%)	37 (68.5%)	45 (72.6%)			
Age	38 ± 11.1	38 ± 10.9	39 ± 11.4	39 ± 8.36	0.732	0.68	0.816
BMI	24.6 ± 4.7	28.7 ± 4.9	29.4 ± 6.1	31.1 ± 6.1	<0.001	<0.001	<0.001
Waist (cm)	85 ± 13.8	95 ± 9.64	98 ± 11.0	103 ± 13.1	<0.001	<0.001	<0.001
BP-S (mmHg)	118 ± 9.3	126 ± 15.9	126 ± 17.3	128 ± 15.1	<0.001	0.003	0.024
BP-D (mmHg)	77 ± 8.2	85 ± 12.6	85 ± 15.0	86 ± 12.7	<0.001	0.004	0.042
Diabetes							
Negative	74 (91.4%)	54 (85.7%)	46 (85.2%)	54 (87.1%)	0.314	0.642	1
Positive	7 (8.6%)	9 (14.3%)	8 (14.8%)	8 (12.9%)			
US fatty liver							
Normal	39 (48.1%)	11 (17.5%)	6 (11.1%)	5 (8.1%)	<0.001	<0.001	<0.001
Intermediate	28 (34.6%)	25 (39.7%)	12 (22.2%)	7 (11.3%)			
Moderate	10 (12.3%)	22 (34.9%)	26 (48.1%)	21 (33.9%)			
Severe	4 (4.9%)	5 (7.9%)	10 (18.5%)	29 (46.8%)			
SWE (m/s)	1.00 ± 0.0601	1.06 ± 0.0552	1.12 ± 0.0645	1.16 ± 0.115	<0.001	<0.001	<0.001
MRI-PDFF (%)	3.6 ± 1.4	8.3 ± 1.7	13.9 ± 1.7	24.6 ± 4.9	<0.001	<0.001	<0.001
AST (U/L)	19 ± 5.4	24 ± 9.5	32.5 ± 20.4	44.4 ± 24.0	<0.001	<0.001	<0.001
ALT (U/L)	20 ± 15.5	34 ± 24.3	54 ± 45.9	84 ± 47.0	<0.001	<0.001	<0.001
GGT (U/L)	26 ± 22.6	39 ± 25.0	50 ± 34.5	57 ± 28.9	<0.001	<0.001	<0.001
ALP (U/L)	67 ± 19.4	77 ± 18.3	76 ± 21.0	80 ± 19.0	<0.001	0.005	0.013
PLT (10^9^/L)	250 ± 55.7	244 ± 59.4	260 ± 63.1	249 ± 46.5	0.919	0.366	0.756
TB (μmol/L)	14.4 ± 6.1	12.2 ± 5.0	14.2 ± 6.7	13.3 ± 4.5	0.138	0.637	0.65
ALB (g/L)	45 ± 3.0	46 ± 2.8	46 ± 3.2	46 ± 3.7	0.008	0.035	0.173
Blood glucose (mmol/L)	5.2 ± 1.0	5.7 ± 1.3	5.6 ± 1.3	5.9 ± 1.4	0.001	0.028	0.032
GHb (%)	6.0 ± 0.9	6.5 ± 1.4	6.6 ± 1.6	7.0 ± 2.0	<0.001	0.004	0.016
TG (mmol/L)	1.2 ± 1.0	2.1 ± 1.3	2.5 ± 1.8	2.4 ± 1.3	<0.001	<0.001	0.01
CHOL (mmol/L)	4.8 ± 1.0	5.0 ± 0.9	5.2 ± 1.0	5.0 ± 1.0	0.027	0.12	0.79
HDL (mmol/L)	1.2 ± 0.3	1.0 ± 0.2	1.1 ± 0.2	1.0 ± 0.2	<0.001	0.002	0.001
LDL (mmol/L)	2.9 ± 0.9	3.1 ± 0.7	3.3 ± 0.9	3.4 ± 0.8	0.003	0.001	0.017
APRI	0.08 ± 0.02	0.10 ± 0.04	0.13 ± 0.09	0.18 ± 0.10	<0.001	<0.001	<0.001
FIB-4	0.74 ± 0.30	0.72 ± 0.28	0.71 ± 0.30	0.78 ± 0.29	0.926	0.668	0.217
NFS	−29.9 ± 2.4	−30.3 ± 2.3	−30.7 ± 2.7	−30.7 ± 2.9	0.047	0.064	0.316

ALB, Albumin; ALP, Alkaline Phosphatase; ALT, Alanine Aminotransferase; APRI, AST to Platelet Ratio Index; AST, Aspartate Aminotransferase; BMI, Body Mass Index; BP-D, Blood Pressure Diastolic; BP-S, Blood Pressure Systolic; CHOL, Total Cholesterol; Cr, Creatinine; FIB-4, Fibrosis-4 Score; GGT, Gamma-Glutamyl Transferase; GHb, Glycated Hemoglobin (HbA1c); HDL, High-Density Lipoprotein; LDL, Low-Density Lipoprotein; MRI-PDFF, MRI-proton Density Fat Fraction; NFS, NAFLD Fibrosis Score; PLT, Platelet Count; SWE, Shear Wave Elastography; TB, Total Bilirubin; TG, Triglycerides; UA, Uric Acid

# , G0 verses G1-3,

$ , G0-1 verses G2-3;

*, G0-2 verses G3

**Fig 2:**
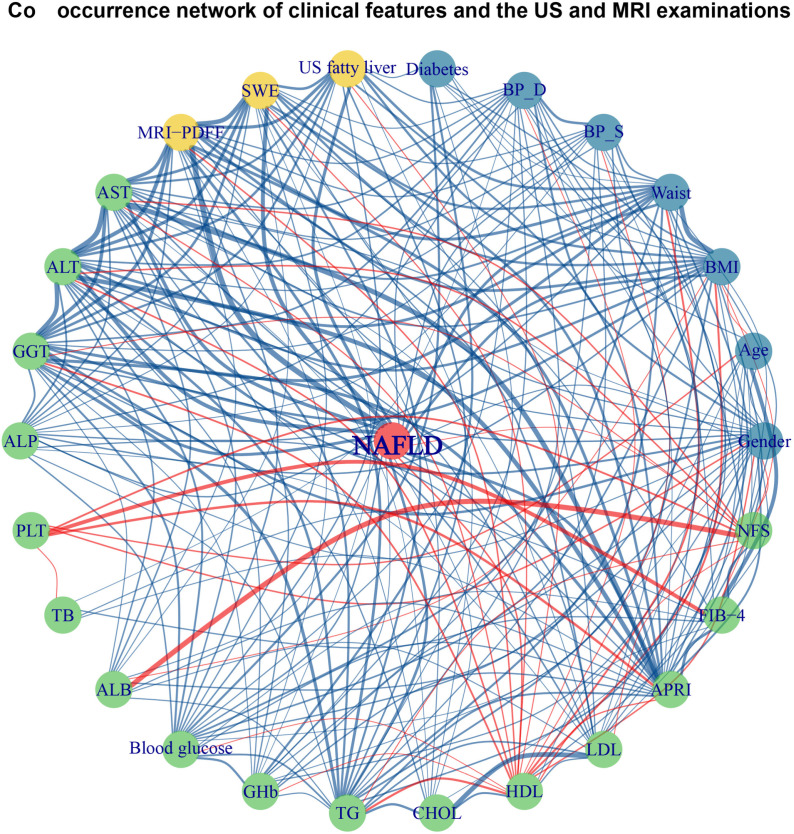
Co-occurrence network of clinical features and ultrasound and MRI examinations in NAFLD. The central node represents NAFLD, with various clinical parameters, laboratory markers, and imaging findings connected through correlation networks. Nodes are color-coded: green for biochemical markers, blue for demographic and metabolic parameters, and yellow for imaging-based features. The network visualization highlights relationships between clinical and imaging markers, with blue lines indicating positive correlations and red lines representing negative correlations. Stronger connections are indicated by thicker lines.

Significant differences were observed across the groups in body mass index (BMI), waist circumference, systolic pressure and diastolic pressure. As the degree of liver steatosis increased, BMI, waist circumference, systolic pressure and diastolic pressure also increased significantly. The serum levels of liver enzymes, including ALT, AST, GGT and ALP, increased progressively with the severity of liver steatosis. Significant differences were also found between groups in fasting blood glucose levels, glycated hemoglobin, triglycerides, HDL and LDL. No significant differences were observed in PLT, TB between groups. Co-occurrence network of clinical features and ultrasound and MRI examinations in NAFLD is shown in Fig 2.

The APRI score increased with the severity of steatosis. Similarly, the FIB-4 index also demonstrated a significant increase as liver steatosis became more severe. However, no statistically significant differences were shown. The NFS values showed minimal variation between groups but were still statistically significant.

### Ultrasound examination and SWE

**Fig 3:**
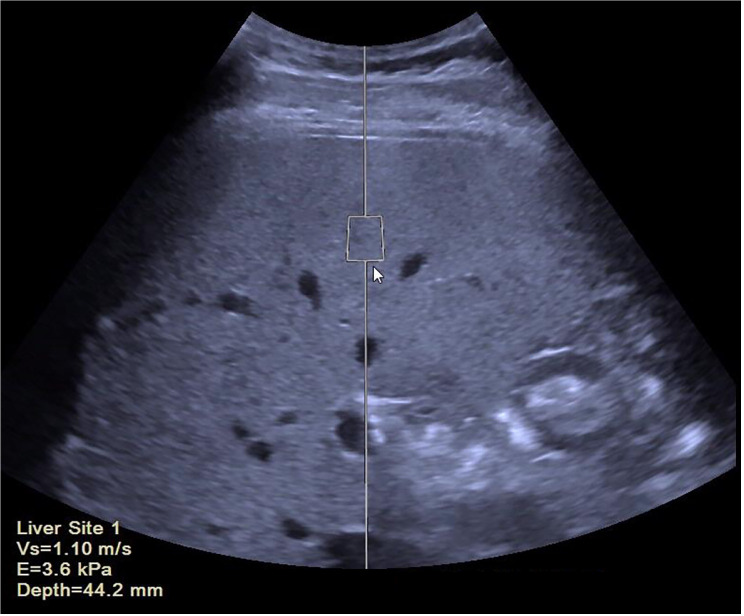
Schematic diagram of liver SWE examination. The SWE velocity (Vs, SWE) is 1.10 m/s, and the transformed SWE elasticity (E) is 3.6 kPa, measured at a depth of 44.2 mm. The region of interest (ROI) is indicated by the square box in the liver parenchyma, avoiding large vessels and bile ducts. This measurement is used for assessing liver stiffness, which is relevant for evaluating liver steatosis in NAFLD.

A schematic diagram of liver SWE examination is shown in Fig 3. The distribution of fatty liver severity on visual ultrasound showed a significant progression in fat accumulation as steatosis severity increased. In the G0 group, 56.8% had normal liver appearance on conventional ultrasound, while this dropped to 17.5% in G1, 11.1% in G2, and 8.1% in G3. The proportion of moderate and obvious fatty liver increased with the severity of steatosis, with G3 showing 46.8% of patients with obvious fatty liver. SWE and showed significant increases with liver steatosis severity. The SWE values increased from 1.00 ± 0.06 m/s in G0 to 1.16 ± 0.15 m/s in G3.

### Stability analysis of SWE

For SWE the ICC values for the groups were as follows: 0.91 for G0, 0.80 for G1, 0.89 for G2, and 0.94 for G3, with ICC values consistently above 0.80 and the highest ICC value observed in the G3 group.

### Correlation analysis between SWE and MRI-PDFF

**Fig 4:**
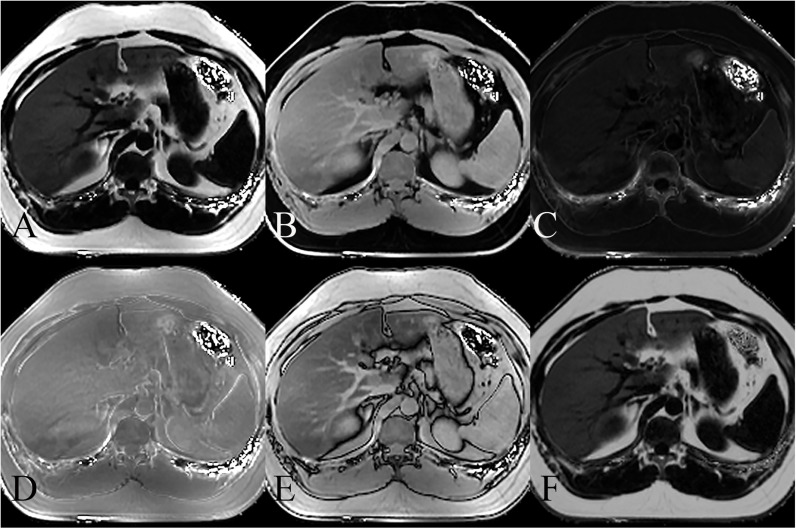
MRI images displaying various sequences used for MRI-PDFF measurement of NAFLD. (A) Fat image, showing the fat distribution in the liver. (B) Water image, highlighting the water content in the liver. (C) R2* map, representing the tissue relaxation rate. (D) In-phase water-fat imaging, where water and fat signals are in phase. (E) Out-of-phase water-fat imaging, showing water and fat signals out of phase. (F) Fat fraction map, quantifying the liver fat content.

A schematic diagram of liver MRI-PDFF examination is shown in Fig 4. Only mild correlation was found between MRI-PDFF and fatty liver evaluated on conventional US (r = 0.57, p < 0.001). For all patients, the correlation coefficient between SWE and MRI-PDFF was 0.65 (p < 0.001). For the separate G0, G1, G2 and G3 groups, the correlation coefficient between SWE and the degree of hepatic steatosis in each group was 0.68, 0.71, 0.68 and 0.53 (all p < 0.001), respectively.

### Diagnostic performance of conventional ultrasound and SWE for grading NAFLD

For conventional ultrasound, the diagnostic performance in differentiating G0 and G1-3 showed an AUC of 0.77 (95% CI: 0.71–0.83) with sensitivity, specificity, PPV and NPV of 0.63, 0.83, 0.89, and 0.50, respectively. The performance for distinguishing G0-1 and G2-3 showed an AUC of 0.76 (95% CI: 0.71–0.82), with sensitivity, specificity, PPV and NPV of 0.74, 0.72, 0.77, and 0.68, respectively. The G0-2 versus G3 comparison showed an AUC of 0.76 (95% CI: 0.70–0.83), with sensitivity, specificity, PPV and NPV of 0.81, 0.61, 0.91, and 0.39, respectively.

For SWE, the diagnostic performance in differentiating G0 and G1-3 showed an AUC of 0.88 (95% CI: 0.84–0.92), with sensitivity, specificity, PPV and NPV of 0.66, 0.94, 0.56, and 0.96, respectively. The performance for distinguishing G0-1 and G2-3 showed an AUC of 0.86 (95% CI: 0.81–0.90), with sensitivity, specificity, PPV and NPV of 0.69, 0.86, 0.78, and 0.80, respectively. The G2 versus G3 comparison showed an AUC of 0.81 (95% CI: 0.75–0.87), with sensitivity, specificity, PPV and NPV of 0.84, 0.64, 0.93, and 0.42, respectively.

**Fig 5:**
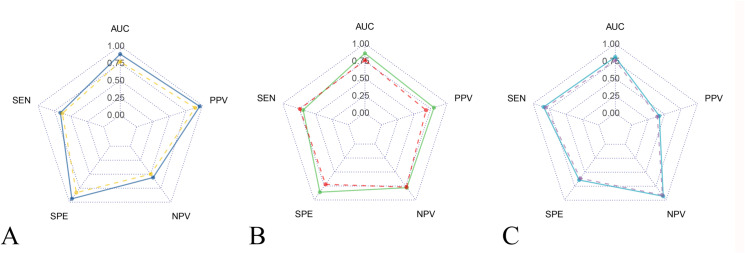
Radar plots illustrating the diagnostic performance of conventional ultrasound and SWE in grading NAFLD. (A) Radar plot represents comparisons of diagnostic performance in G0 vs G1-G3 for conventional ultrasound (yellow) and SWE (blue). (B) Radar plot represents comparisons of diagnostic performance in G0 vs G1-G3 for conventional ultrasound (red) and SWE (green). (B) Radar plot represents comparisons of diagnostic performance in G0 vs G1-G3 for conventional ultrasound (purple) and SWE (blue). Key diagnostic metrics displays area under the curve (AUC), sensitivity (SEN), specificity (SPE), positive predictive value (PPV), and negative predictive value (NPV). Higher values indicate better diagnostic accuracy.

The SWE showed better diagnostic performance in G0 versus G1-3 (p = 0.003), G0-1 versus G2-3 (p = 0.002), but not in G0-2 versus G3 (p = 0.262), by DeLong tests (Table 2 and Fig 5).

**Table 2:** The diagnostic performance of conventional ultrasound and SWE.

	AUC	95%CI	SPE	SEN	NPV	PPV
US G0 versus G1-3	0.77	0.71–0.83	0.63	0.83	0.50	0.89
US G0-1 versus G2-3	0.76	0.71–0.82	0.74	0.72	0.77	0.68
US G0-2 versus G3	0.76	0.7–0.83	0.81	0.61	0.91	0.39
SWE G0 versus G1-3	0.88	0.84–0.92	0.66	0.94	0.56	0.96
SWE G0-1 versus G2-3	0.86	0.81–0.90	0.69	0.86	0.78	0.80
SWE G0-2 versus G3	0.81	0.75–0.87	0.84	0.64	0.93	0.42

AUC, Area Under the Curve; CI, Confidence Interval; SEN, Sensitivity; SPE, Specificity; NPV, Negative Predictive Value; PPV, Positive Predictive Value; US, Ultrasound

## Discussion

This study explored the feasibility of using SWE to assess NAFLD, comparing its results with MRI-PDFF as the gold standard for measuring liver fat content. We found that SWE demonstrated a reliable and consistent ability to assess liver steatosis across different stages of NAFLD without fibrosis.

Our analysis revealed that the correlation between SWE and MRI-PDFF was statistically significant only in patients with mild and moderate hepatic steatosis. This finding is consistent with previous study reported a moderate correlation between SWE and severity of NAFALD in the early stages of liver steatosis but found this correlation weakened in more advanced stages of the disease [16]. The result indicated that the reliability of SWE in detecting changes in liver fat content was diminished as steatosis became more severe. This likely dues to the complex interplay between fat accumulation and liver fibrosis [17].

Previous studies emphasized that SWE and MRI-PDFF are valuable for diagnosing NAFLD and assessing its progression. However, their performance varies across different stages of liver disease [8,18]. Tahmasebi et al. found that while SWE and MRI-PDFF showed a positive correlation in liver fibrosis, the relationship between these methods and liver fat quantification remained inconclusive for severe cases [19]. Similarly, Kaposi et al. demonstrated that while ultrasound-based methods can estimate liver fat content, the correlation with MRI-PDFF remained strong in early stages but weakened in later stages of the disease [20]. These findings reinforce the notion that while SWE has reliability in assessing liver stiffness, it may struggle to track hepatic fat in more severe stages of NAFLD due to fibrosis existing. The findings of this study highlight the superior diagnostic performance of SWE compared to conventional ultrasound in assessing the grade of liver steatosis in early-stage NAFLD. This is consistent with previous research, such as the study by Selvaraj et al., which also found that SWE outperforms conventional ultrasound in the quantification of liver steatosis, particularly for lower stages of the disease [21].

The results from SWE measurements in our study showed that SWE were found to be relatively stable across all grades of steatosis, with increases in velocity as steatosis increased. This stability suggests that SWE can be sensitive enough to detect early changes in liver fat content [22]. The ICC values obtained for SWE in our study were excellent across all groups, which demonstrates the high reproducibility and consistency of SWE as a tool for assessing liver steatosis. This is particularly important in clinical settings where repeated monitoring of NAFLD is required. Results showed that as SWE can offer both higher sensitivity and accuracy compared to conventional ultrasound. Moreover, the results from this study suggest that SWE is more effective in identifying early stages of liver steatosis, demonstrated SWE’s potential in providing a more reliable, reproducible assessment of liver fat content in the early-stage of NAFLD [23]. The good correlation in SWE and MRI-PDFF suggests potentially reducing the need for more invasive procedures like liver biopsy.

However, the study also observed that there was no significant difference between SWE and conventional ultrasound in differentiating between the more advanced stages of liver steatosis (G0-2 versus G3). Previous study reported that the performance of SWE and conventional ultrasound may converge in distinguishing between the more severe stages of liver steatosis, possibly due to the limited dynamic range of both techniques in advanced liver disease. Thus, while SWE provides enhanced diagnostic accuracy, particularly for early-stage NAFLD, its role in advanced disease might not significantly surpass conventional ultrasound.

Despite promising findings, several limitations exist. The study’s cross-sectional design limits causal conclusions and the assessment of disease progression. Thus, further longitudinal studies is needed. Additionally, SWE relies on operator skill, which can introduce variability, influenced by factors such as patient positioning. While MRI-PDFF is widely validated as a non-invasive surrogate to histology-determined steatosis, the absence of liver biopsy may reduce the definitive rigor of our study. Future studies incorporating histological assessment would strengthen these findings. Furthermore, SWE shows only modest correlation with MRI-PDFF in quantifying hepatic steatosis. This moderate correlation limits the diagnostic confidence when using SWE alone for detecting subtle changes in fat content or for accurate staging in early NAFLD. Future studies should explore further approaches that complement SWE with to improve the diagnostic accuracy. Finally, the lack of detailed assessment of liver fibrosis or cirrhosis limits our understanding of their interaction with liver fat and the observed weak correlation in advanced stages of NAFLD.

## Conclusion

In conclusion, SWE is a reliable tool for assessing liver steatosis in early-stage NAFLD without fibrosis. The ability of SWE can be used to accurately measure liver fat, but which might be limited particularly in advanced stages. SWE’s reproducibility makes it valuable for monitoring steatosis.
